# Transformation of Pectins into Non-Ionic or Anionic Surfactants Using a One-Pot and Cascade Mode Process

**DOI:** 10.3390/molecules26071956

**Published:** 2021-03-31

**Authors:** Damien Milliasseau, Jelena Jeftić, Freddy Pessel, Daniel Plusquellec, Thierry Benvegnu

**Affiliations:** 1 Univ. Rennes, CNRS, ISCR-UMR 6226, Ecole Nationale Supérieure de Chimie de Rennes, F-35000 Rennes, France; damien.milliasseau@orange.fr (D.M.); jelena.jeftic@ensc-rennes.fr (J.J.); 2 SurfactGreen, 11 allée de Beaulieu, CS 50837, CEDEX 7, F-35708 Rennes, France; freddy.pessel@surfactgreen.com

**Keywords:** galacturonates, pectins, cascade process, polysaccharide depolymerisation, surfactants, readily biodegradability, non-ecotoxicity

## Abstract

The present article describes the one-pot synthesis of double- and single-tailed surfactants by a cascade process that involves the hydrolysis/butanolysis of pectins into butyl galacturonate monosaccharides followed by transesterification/transacetalisation processes with fatty alcohols, and subsequent aqueous basic and acid treatments. The cascade mode allows the depolymerisation to proceed more efficiently, and the purification conditions are optimised to make the production of single-tailed surfactants more manufacturable. These products in a pure form or as mixtures with alkyl glycosides resulting from butanolysis and transglycosylation of pectin-derived hexoses, exhibit attractive surface-tension properties, especially for the *n*-oleyl ᴅ-galactosiduronic acid products. In addition, a readily biodegradability and an absence of aquatic ecotoxicity are shown for the galacturonic acid derivatives possessing an oleyl alkyl chain at the anomeric position.

## 1. Introduction

**Figure 1:**
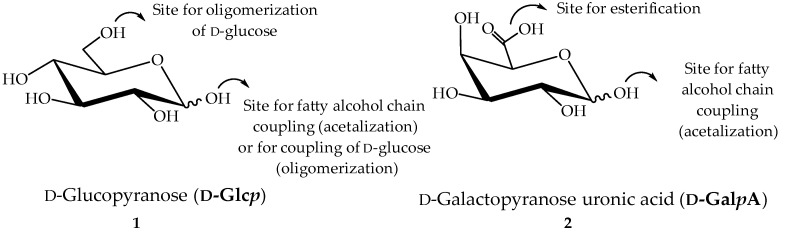
Structure of ᴅ-glucopyranose (ᴅ-Glc*p*) 1 and ᴅ-galactopyranoside uronic acid (ᴅ-Gal*p*A) 2 as starting materials for surfactant synthesis.

With the focus on reducing dependency on fossil fuel resources, carbohydrate-based surfactants are an important class of amphiphilic compounds and the most representative alkylpolyglucosides (APGs) [1,2] that are currently widely present in the world market (about 85,000 t/year in 2010 [1,2]) of neutral surfactants. Initially obtained by reacting fatty alcohols with dextrose (ᴅ-glucose) syrup or starch via a Fischer reaction [3], there is no doubt that the preferred process to produce APGs is to directly transform cellulose using a one pot procedure (Figure 1). Unfortunately, the hydrolysis of the robust crystalline structure of cellulose [4] remains highly challenging. Nevertheless, the research field of cellulose valorisation has become very popular in the last decade and recent papers describe one-pot multistep processes to transform cellulose or other neutral polysaccharides such as xylan or agarose, into alkylglucosides as non-ionic surfactants [5,6,7,8,9,10,11,12,13,14,15].

**Scheme 1:**
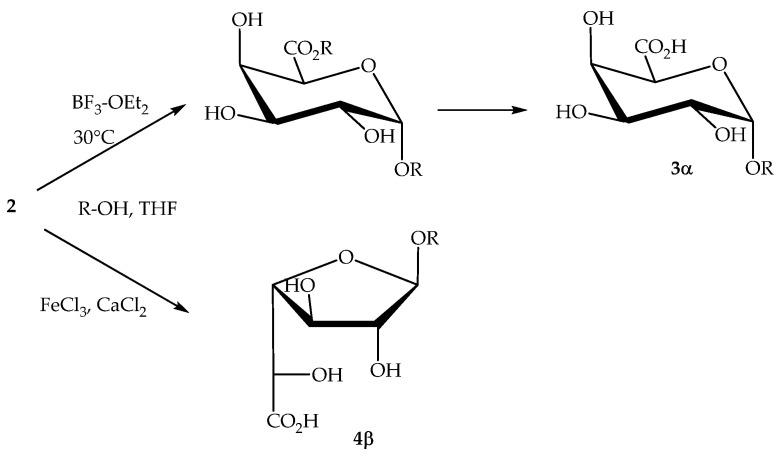
Selective transformation of 2 into alkyl ᴅ-galactosiduronates.

However, anionic surfactants, carrying a negative charge on the polar head, are the ‘traditional’ ones and they remain the commercially most important family of surfactants [16]. The properties of these anionic compounds have been well explored and are highly dependent of the alkyl chain length of their hydrophobic part: wetting agents (C8–C10); detergents (C12–C16); emulsifiers and softeners (C18–C22) [17]. We and others have developed inter alia a new synthesis of alkyl ᴅ-galacturonates directly from unprotected ᴅ-galacturonic acid 2. *O*-Glycosidation of totally unprotected 2 in THF provided α-pyranosides 3α when promoted with BF_3_.OEt _2_ at 30 °C, whereas β-furanosides 4β were obtained in the presence of FeCl_3_ as promotor and CaCl_2_ as complexing agent (Scheme 1) [18,19,20].

Nevertheless, as in the case of neutral carbohydrates, it would be highly desirable to find a process producing alkyl ᴅ-galacturonic acids from natural polymers, e.g., pectins. Pectins and pectic acids are the major macromolecules present between the cell and the primary cell wall of plants and they contribute to various cell functions, including cell wall integrity, defence, signalling and cell adhesion. Historically, only polymers rich in ᴅ-galacturonic acid were classified as pectins [21].

Despite a wide structural diversity, pectins can be described as a multi-domain structure [22,23]:

A pectic backbone is a homo-polymer of (1-4)-α-ᴅ-Gal*p*A (HGA) with a varying degree of methyl-esterification and of acetylation of Gal*p*A at the 2 and 3 positions. Within the HGA region, a persistent length occurs for about 15 monosaccharide residues. This sequence alternates with apiogalacturonans. The chain is interrupted by moieties of alternating -2)-α-Rha-(1-4)-α-Gal*p*A-(1-, named rhamnogalacturonan RGI. This portion is highly branched and the side branches are mostly composed of galactans, arabinans and arabino galactans linked to HGA through a rhamnose moiety.

Complex oligosaccharides could be attached to HGA at 2 or 3 positions of Ga*lp*A and form the rhamnogalacturonan RG II domain of pectins [22,23].

Pectins are highly heterogeneous polysaccharides and differ from species to species in the nature of side chains and in the degree of methylation and acetylation of Gal*p*A, but all species contain in addition to Gal*p*A, a lot of neutral carbohydrates such as ʟ-rhamnose, ᴅ-galactose, ᴅ-xylose, etc., [22,23]. In the present work, we used low methylated pectins (DM ≤ 50%) extracted from lemon peel (*Citrus lemonia*) and provided by the chemical CARGILL Company (Redon, France). Acid hydrolysis is most commonly used for pectin hydrolysis and can be performed with H_2_SO_4_, HCl, or TFA, etc., usually at concentration 1–2 M and at temperatures of 100–120 °C [24]. The disadvantage of this method is that glycosidic bonds have different susceptibilities to acids and that results in their gradual hydrolysis. The combination of chemical or physical pretreatments followed by acid or enzymatic treatments, may be useful in isolation and structure elucidation of pectic polysaccharides [25]. As an example, citric acid favours the solubilisation of pectins in hot water [25]. To our knowledge ultrasound-assisted hydrolysis does not affect the chemical structures of oligomers [26] but enzymatic hydrolysis is generally performed using a combination of pectin lyase and (endo)-polygalacturonase and therefore provides predominantly unsaturated and methyl-esterified oligomers of ᴅ-Gal*p*A [25].

Within this context, the main challenges of this research were (i) to find a method of pectin hydrolysis that enables complete depolymerisation while maintaining chemical structures and properties of obtained monomers, and therefore using directly the polysaccharide as the starting material, (ii) to find an intermediate reference mark (a new platform molecule) in order to determine quantitatively the degree of depolymerisation of the polysaccharide and the intermediate oligosaccharides, (iii) to finalise a one pot process without requiring the isolation and the purification of reaction intermediates and (iv) to propose eco-friendly conditions using biocompatible and/or recyclable reagents without waste production. The non-ionic and anionic surfactants produced through these procedures will be evaluated in terms of surface activities, biodegradability and ecotoxicity.

## 2. Results

### 2.1. Characterisation of Pectin Starting Material

**Figure 2:**
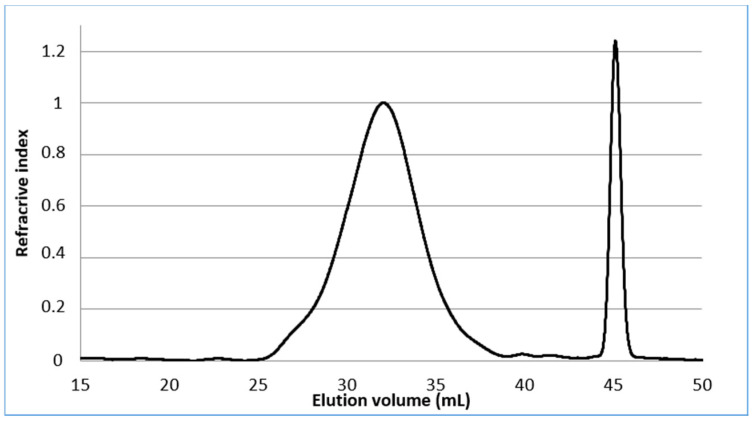
HPSEC analysis of pectin.

**Figure 3:**
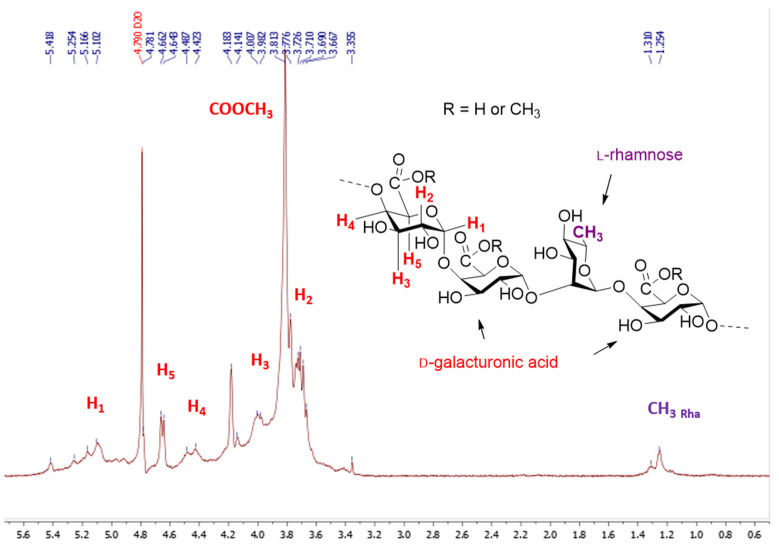
^1^H NMR (400 MHz) spectrum in D_2_O of the pectin.

The molecular weights of the starting material were measured by HPSEC. The pectin showed (Figure 2) a broad distribution of molecular weights eluting at the void volume of the column (Mw: 381 kDa) and had a high polydispersity index of about 3.8. The ᴅ-galacturonic content determined by a colorimetric method [27] using *m*-hydroxydiphenyl, is in the range 66.9% in mass percent and the degree of methyl esterified **GalA** determined by the usual conductimetric method [28] was found to be 30.2%, expressed as moles of methyl esters per 100 mol of **GalA**. The neutral sugar content of pectin was determined by gas chromatography after methanolysis of the crude product and trimethylsilylation of the hydrolysate and using myo-inositol as an internal standard [29,30]. Our results show that only 61.3% of the pectin was transformed into monosaccharides under the present conditions and that the sample is made up of **GalA** (49.9%) and 11.4% neutral sugars. Among the neutral sugars, galactose (7.1%) was the major sugar, followed by rhamnose (2.4%) and glucose (1.9%). Our pectin sample was therefore composed of **GalA**, **Gal**, **Rha** and **Glc** with peaks molar ratio of 1:0.15:0.06:0.05 (Figure S1, supplementary materials). Finally, ^1^H NMR spectra of pectin in D_2_O (Figure 3) were in agreement with literature data [31] except for the signal assigned to H-5 as a narrow doublet at 4.68 ppm instead of a broad singlet at 5.05 as reported in reference [31].

### 2.2. Synthesis of Galacturonate Derivatives

The synthesis of galacturonic acid-based surfactants possessing one or two alkyl chains was achieved through the preparation of *n*-butyl uronate monosaccharide intermediates involving methanesulfonic acid-promoted hydrolysis/butanolysis of pectin, followed by in situ transesterification and transglycosylation reactions with fatty alcohols to provide the neutral double-tailed surfactants. An additional saponification step was required to give access to the single-tailed surfactants.

#### 2.2.1. Direct Transformation of Pectin into *n*-Butyl (*n*-butyl ᴅ-galactosiduronates) C_4_GalC_4_

**Figure 4:**
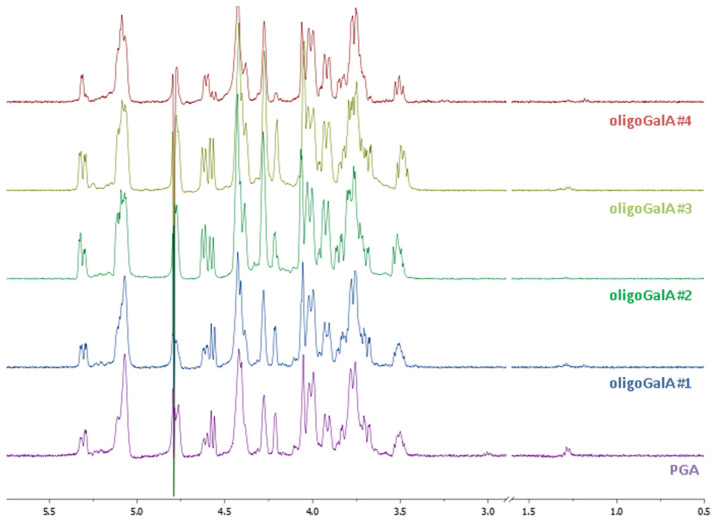
^1^H NMR (400 MHz) spectra in D_2_O of polygalacturonic acid (PGA) and oligo-galacturonic acids. **PGA**: 1N HCL, 100 °C, 5 h; **OligoGalA#1**: 1N HCL, 100 °C, 5 h then 0.01N HCl, 120 °C, 40 min; **OligoGalA#2**: 1N HCL, 100 °C, 5 h then 0.01N HCl, 120 °C, 4 h; **OligoGalA#3**: 1N HCL, 100 °C, 5 h then 1N HCl, 120 °C, 40 min; **OligoGalA#4**: 1N HCL, 100 °C, 5 h then 1N HCl, 120 °C, 4 h.

The conventional process for hydrolysis of pectins involves the use of aqueous mineral acids at elevated temperatures and pressures. In this paper, the main factors influencing the hydrolysis of pectin were investigated including the nature of the acid and the effect of the acidic dosage, the reaction temperature and time, the content of water, the use of an additive in order to trap the monomers and the oligomers formed in the reaction. Concentrated sulfuric acid was effective in the depolymerisation process but reacted with the primary alcohol additive to give the corresponding alkylsulpate as a main product. We then investigated a second approach using aqueous HCl. After experimentation, we found that a first step using 1N HCl at 100 °C for 5 h gives after neutralisation with sodium hydroxide and precipitation with isopropanol and centrifugation, a soluble phase composed of neutral carbohydrates (Rh, Gal and Glc) and of some oligomers of **ᴅ-GalpA** and an insoluble polygalacturonic acid (**PGA**). The second step was performed on the **PGA** in 1N HCl at 120 °C for 40 min. or 4 h. The resulting oligo-Galp A were analysed by ^1^H NMR (Figure 4) and by HPSEC and were characterised respectively to be the nonamers (Mw: 1600 g mol^−1^; Mw/Mn: 1.25: **OligoGalA#1;** Mw: 1600 g mol^−1^; Mw/Mn: 1.60: **OligoGalA#3**), the decamers (Mw: 1800 g mol^−1^; Mw/Mn: 1.46: **OligoGalA#4**) and the pentadecamers (Mw: 2600 g mol^−1^; Mw/Mn: 1.27: **OligoGalA#2**) of **ᴅ-GalA**. These results do not clearly demonstrate the influence of the HCl concentration and the depolymerisation time on the hydrolysis efficiency, but they show that a minimal polymerisation degree of 9 can be obtained.

**Scheme 2:**
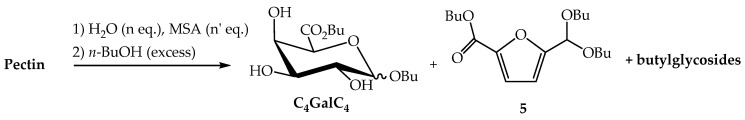
Transformation of pectin into *n*-butyl (*n*-butyl ᴅ-galactosiduronates) C_4_GalC_4_.

In a third approach, we found that alkyl sulfonates, such as methyl-, camphor- and *p*-toluene-sulfonic acids were effective to promote the hydrolysis of pectin and in the present work, we privileged methanesulfonic acid (MSA) which is well-known for its environmental benefits and is considered as a biodegradable and low toxic product [32]. In order to determine the degree of depolymerisation of pectin and of polyGalpA, trials indicated that a partial hydrolysis (5 h) was required before introducing the alcohol to perform the Fischer reaction. After experimentation, we decided to carry out the introduction of the fatty alcohol on the sugar by transesterification and transacetalisation of the previously prepared *n*-butyl derivatives. Our main results are summarised in Scheme 2 and in Table 1. To a suspension of pectin in water (*n* eq.), was added MSA (*n*′ eq.) and the mixture was heated under reflux at atmospheric pressure for 5 h. An excess of *n*-butanol was then added and the mixture was heated under reflux in order to eliminate the water initially added and the water formed during the esterification/glycosylation reactions, by using a Dean-Stark apparatus. A variety of conditions were tried in order to enhance the yield of monomeric butyl ᴅ-galacturonic acid derivatives C_4_GalC_4_. After neutralisation and work-up, the organic residue was subjected to silica gel column chromatography that allowed the separation of a mixture of butyl (butyl ᴅ-galactosiduronates) C_4_GalC_4_, from the butyl 5-(dibutoxymethyl)-2-furanoate 5 and the minority butyl glycosides derived from neutral sugars present in the starting material. Compounds C_4_GalC_4_ were isolated as expected as mixtures where the α-pyranosidic and the β-furanosidic forms were largely prevalent [18,19,20]. The first step of the process was generally performed in 1000 eq. of water for 5 h (Table 1; entries 1–4) and in the presence of 2.5 eq. of MSA. Indeed, lowering the quantity of water and/or of MSA (entries 5–7) had a drastic influence on the yield of expected glycoside-esters C_4_GalC_4_. We then attempted to minimise the yield of the acetal ester 5 of 2,5-formylfurancarboxylic acid [33,34], which could be produced by dehydration of monomeric ᴅ-galacturonic acid 2 or of its derivatives. When the second step of the reaction was performed at reflux of butanol, the yield of the by-product 5 attained 27% after 24 h and 36% after 48 h (entries 1,2). However, when the temperature of the medium was maintained at 80 °C the yield of the by-product fell drastically (entries 4–7). Finally, the best results were obtained under the conditions described in entry 4, and the expected compounds C_4_GalC_4_ were isolated in an excellent 76% yield.

#### 2.2.2. Direct Transformation of Pectin into Double-Tailed Surfactants

**Scheme 3:**
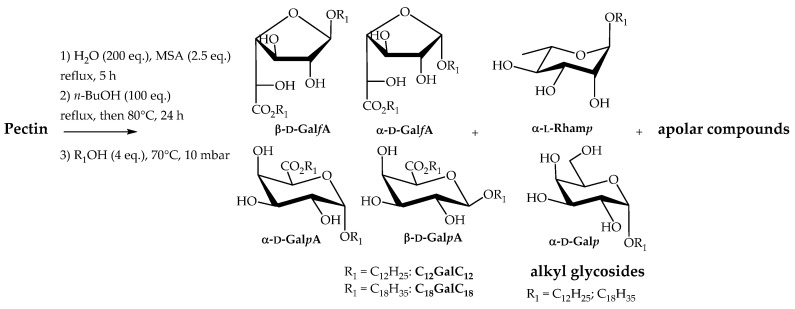
Transformation of pectin into *n*-alkyl (*n*-alkyl ᴅ-galactosiduronates) C_n_GalC_n_ and alkyl glycosides.

At this stage, we investigated the preparation of nonionic surfactants possessing two similar fatty chains, directly from pectin through a one-pot process that involved the pectin hydrolysis/butanolysis followed by transesterification and transglycosylation reactions with fatty alcohols (Scheme 3). Dodecyl and oleyl alcohols were selected as the fatty alcohols since they bring a lipophilic character to the glycosides suitable for applications as emulsifiers. This strategy offers the opportunity to provide *n*-alkyl (*n*-alkyl ᴅ-galactosiduronate) surfactants **C_n_GalC**_**n**_ as the major products and alkyl glycosides derived from ʟ-rhamnose, ᴅ-galactose or ᴅ-glucose as the minor products. Indeed these minority constituents of the pectin are also involved in butanolysis and transacetalisation reactions to give the corresponding long-chain alkyl glycosides. The transformation of pectin into dodecyl or oleyl monosaccharide surfactants has been carried out first by hydrolysing pectin with water (200 eq.) in the presence of 70% MSA (2.5 eq.). Even if these conditions (**entry 5**, Table 1) did not afford the best yield for the production of *n*-butyl (*n*-butyl ᴅ-galactosiduronates) **C_4_GalC_4_**, we made the choice to minimise the quantities of solvents used to facilitate an industrial development of the process. The reaction mixture was heated under reflux for 5 h. *n*-Butanol (100 eq.) was then added and the water, which was initially added in the reaction mixture and formed during the reaction, was eliminated by using a Dean-Stark apparatus. The temperature was cooled to 80 °C and the reaction mixture was stirred for 24 h before adding fatty alcohols (4 eq.). The unreacted *n*-butanol initially added and formed during the transacetalisation step was removed at 70 °C and under reduced pressure (10 mbar). At this end of the reaction, the reaction mixture was neutralised with a 1N aqueous solution of NaOH and extractions with EtOAc were achieved. Once the organic phases dried and concentrated, the residue was chromatographed on silica gel column with gradient elution consisting of CH_2_Cl_2_/EtOAc from 100:0 to 50:50. Under these conditions, *n*-dodecyl (*n*-dodecyl ᴅ-galactosiduronate) and *n*-oleyl (*n*-oleyl ᴅ-galactosiduronate) surfactants **C_12_GalC_12_** and **C_18_GalC_18_** were isolated with yields of 33% and 40% respectively. These values are higher than those obtained for the synthesis of *n*-butyl (*n*-butyl ᴅ-galactosiduronates) **C_4_GalC_4_** (21%, **entry 5**, Table 1) which means that hydrolysis and alcoholysis reactions of oligo(poly)saccharides occur even after the addition of fatty alcohols.

The concentrated organic phases isolated before column chromatography were considered as semi-purified mixtures (**C_12_PectC_12_** and **C_18_Pect C_18_**) and their surface activities were also evaluated in addition to purified products (see Section 2.3). These mixtures are composed of residual fatty alcohol (R*f* = 0.75; CH_2_Cl_2_/MeOH (95/5, *v*/*v*)), *n*-alkyl (*n*-alkyl ᴅ-galactosiduronates) **C_n_GalC_n_** (R*f* = 0.5–0.4; CH_2_Cl_2_/MeOH (95/5, *v*/*v*)) and alkyl glycosides (R*f* = 0.3–0.1; CH_2_Cl_2_/MeOH (95/5, *v*/*v*)) in addition to non-identified apolar products (R*f* = 0.99; CH_2_Cl_2_/MeOH (95/5, *v*/*v*)). Four isomers were identified and quantified by NMR studies for *n*-alkyl (*n*-alkyl ᴅ-galactosiduronate) compounds **C_n_GalC_n_**, with β-furanosiduronate (β-ᴅ-Gal*f*A) and α-pyranosiduronate (α-ᴅ-Gal*p*A) forms predominating (Table 2). The composition in mass of the semi purified **C_12_PectC_12_** and **C_18_PectC_18_** were determined after silica gel column chromatography (Table 2). Residual fatty alcohol represents the highest proportion of the mixture followed by the double-tailed surfactants **C_n_GalC_n_**, and alkyl glycosides and non-identified non-polar products present in equivalent amounts. Further investigation based on NMR and mass spectrometry experiments have been carried out in order to determine the structure of alkyl glycosides. In the oleyl series, a second column chromatography separation of the alkyl glycoside fraction using CH_2_Cl_2_/EtOAc/MeOH (20/75/5, *v*/*v*/*v*) as the eluent, highlighted the presence of alkyl α-ʟ-rhamnopyranoside and alkyl α-ᴅ-galactopyranoside isomers (Scheme 3).

#### 2.2.3. Direct Transformation of Pectin into Single-Tailed Surfactants

**Scheme 4:**
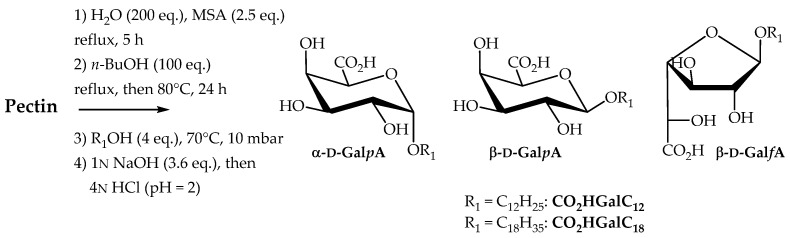
Transformation of pectin into *n*-alkyl ᴅ-galactosiduronic acids **CO_2_HGalC_n_**.

Our next goal was to develop a one-pot procedure to prepare neutral (carboxylic acid form) or anionic (carboxylate salt form) single-tailed surfactants **CO_2_HGalC_n_** and **CO_2_NaGalC_n_**. The same synthetic process as for the preparation of double-tailed surfactants **C_n_GalC_n_** was used followed by an additional saponification step (Scheme 4). Compounds **C_12_GalC_12_** and **C_18_GalC_18_** were therefore saponified (1N aq. NaOH solution, 3.6 eq.) for 12 h at 30 °C to yield the corresponding sodium carboxylates **CO_2_NaGalC_n_**. In order to isolate the galacturonic derivatives and to determine yields, liquid–liquid extraction techniques were carried out. The reaction mixture was taken up in diethyl ether and then washed several times with water to separate the fatty alcohol, degradation products and alkyl glycosides, soluble in the organic phase, from water-soluble galacturonate derivatives **CO_2_NaGalC_n_**, methanesulfonic acid salts and residual oligosaccharides. The aqueous phases were then acidified to pH 2 using a solution of 4N HCl, extracted with EtOAc to furnish the single-tailed surfactants in 37% (**CO_2_HGalC_12_**) and 42% (**CO_2_HGalC_18_**) overall yields. With the aim to provide a semi-purified version of these carboxylic acids, isolated surfactants **CO_2_HGalC_n_** were added to the mixture composed of fatty alcohol, degradation products and alkyl glycosides. The mass composition of these surfactant compositions (semi-purified **CO_2_HPecC_12_** and **CO_2_HPecC_18_**) including the isomeric *n*-alkyl ᴅ-galactosiduronic acid mixture **CO_2_HGalC_n_**, is described in Table 3.

#### 2.2.4. One-Pot Manufacturable Process for the Production of Single-Tailed Surfactants

**Figure 5:**
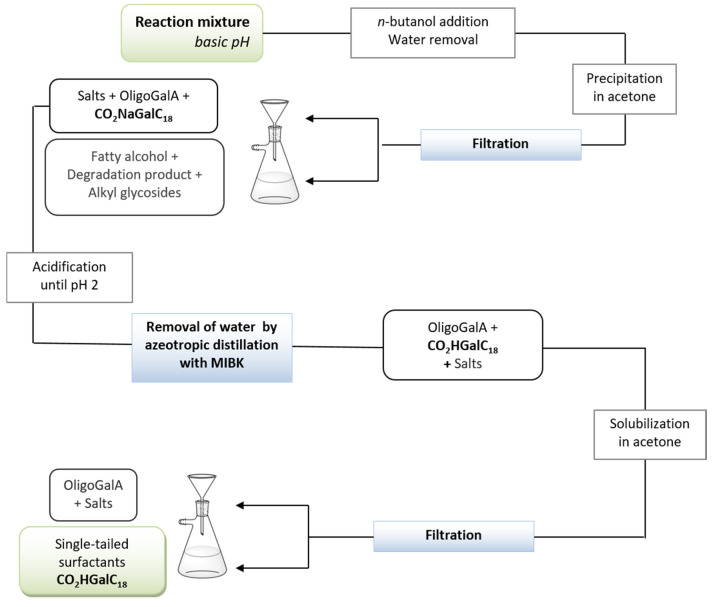
Procedure based on successive filtrations for the isolation of purified *n*-oleyl ᴅ-galactosiduronic acids **CO_2_HGalC_18_**.

Our attention was finally directed towards the development of a process more easily transferable to an industrial scale, based upon the exclusive use of filtration steps to separate the products and to isolate the desired *n*-alkyl ᴅ-galactosiduronic acids **CO_2_HGalC_n_** with a high purity. The study was conducted from 85 g of pectin, using oleyl alcohol as the fatty alcohol for physico-chemical reasons, which is explained in the next section. Thus, the procedure consisted first of a hydrolysis of pectin under reflux for 5 h followed by the esterification and acetalisation reactions in the presence of *n*-butanol to provide access to butyl intermediates. The latter mixture was maintained under reflux for 24 h and the water removal was achieved by using a Dean-Stark apparatus. Oleyl alcohol (5.6 eq.) was subsequently added to the reaction medium and once the double-tailed compounds were obtained (4 h, 70 °C, 16 mbar), an aqueous solution of 1N sodium hydroxide (5 eq.) was finally added to allow the saponification reaction. This reaction mixture was stirred at 70 °C for 5 h and then at room temperature overnight. From the crude reaction mixture at basic pH (Figure 5), water was removed by azeotropic distillation after addition of *n*-butanol. Incorporation of acetone into the water-free mixture resulted in precipitation of sodium *n*-oleyl ᴅ-galactosiduronates **CO_2_NaGalC_18_** in addition to salts and residual oligogalacturonates. Filtrate was composed of residual fatty alcohol, degradation products and alkyl glycosides. The solid was solubilised into water and the solution was treated by an aqueous solution of 5N HCl until pH ~ 2. Water was then eliminated by an azeotropic distillation with methylisobutylketone (MIBK) and a selective solubilisation of *n*-oleyl ᴅ-galactosiduronic acids **CO_2_HGalC_18_** in acetone was carried out. After removal of organic solvents, the single-tailed galacturonic acid derivatives **CO_2_HGalC_18_** were isolated in high purity (>95%) with an overall yield of 62% and an isomeric composition of β-ᴅ-galactofuranosiduronic acid (19% in mol), β-ᴅ-galactopyranosiduronic acid (13% in mol) and α-ᴅ-galactopyranosiduronic acid (68% in mol). This highly efficient process is promising for the industrial production of single-tailed surfactants derived from pectins.

### 2.3. Physico-Chemical Evaluations

At the interface between two phases (liquid–gas or liquid–liquid), a clear transition of polarity can be observed. We speak of surface tension when it comes to liquid systems—gas (usually water–air), and interfacial tension when it comes to two non-miscible liquids (usually oil–water). Because of their amphiphilic character, surfactants have the particularity of positioning themselves preferentially at the interface between the different phases of a system. We thus obtain structures whose polar part, hydrophilic, is immersed in the most polar constituent, usually water, while the non-polar part, hydrophobic, is in a little or no polar environment (air or oil). In general, the presence of surfactants in these systems tends to decrease the tension at the interface. We considered exploring the interfacial tension of a model system, oil–water. The decrease in surface tension is proportional to the concentration until reaching a limit value, which is representative of the saturation of the surface of the medium in which the surfactant is dispersed, or of the interface, according to the system studied. The critical micellar concentration (CMC) corresponds to the concentration value for which the molecules, present in solution, are in sufficient concentration to spontaneously form micelles, or other forms of aggregates. This value is characteristic of a surfactant at a given temperature value. The surfactants synthesised in this work, in their non-ionic form, are soluble only in the oily phase.

#### 2.3.1. Measurements of Interfacial Tension

The interfacial tension measurements were carried out using a tensiometer (Krüss K100) with a Ring of Noüy, at a temperature of 25 °C. The measurements concerned oil-water systems for which the compounds were previously solubilised in the oily phase. The sunflower oil, used in these measurements, is a commercial oil composed of fatty, saturated, generally palmitic (C_16:0_) and stearic (C_18:0_), monounsaturated, oleic acid (C_18:1_) and mostly polyunsaturated, derived from linoleic acid (C_18:3_). The determination of interfacial tension using the ring tensiometer was performed by a method called ‘ring tear-off’. Two phases, watery and oily, are successively introduced to form two layers of superimposed liquids. The ring is positioned under the interface, here in the aqueous phase, of higher density, and then it is removed very slowly until the force, measured using the ring, reaches its maximum. Experimentally, we found that the value of interfacial tension, initially about 25 mN m^−1^ for surfactant-free systems, gradually decreases to a constant level. The value of surfactant properties was determined taking into account the measured minimum tension and galacturonic derivative concentrations. Measurements were carried out on purified compounds and then on semi-purified mixtures isolated before the final purification step.

**Figure 6:**
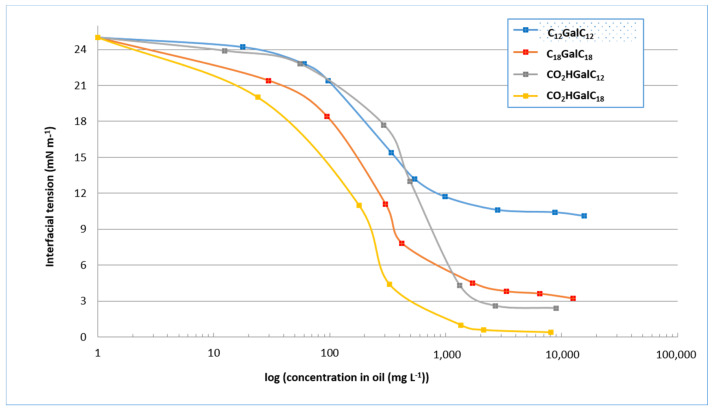
Values of interfacial tension of purified compounds **C_n_GalC_n_** and **CO_2_HGalC_n_**.

Determination of the interfacial tension curves of purified single and double-tailed compounds (Figure 6) was used to evaluate the minimum tension values. As we can deduce from the experiments, they are on the order of 11 mN^−1^ for the purified compounds **C_12_GalC_12_** at a concentration of approximately 1 g^−1^. The tension values are on the order of 5 mN m^−1^ for the purified compounds **C_18_GalC_18_** at a concentration of approximately 900 mg L^−1^. They are on the order of 3 mN m^−1^ for the purified compounds **CO_2_HGalC_12_** at a concentration of approximately 1.05 g L^−1^, and on the order of 1 mN m^−1^ for the purified compounds **CO_2_HGalC_18_** at a concentration of approximately 800 mg L^−1^. These results demonstrate, on the one hand, that oleyl single or double-tailed compounds decrease the interfacial tension to reach minimum values lower than their equivalents containing lauryl chains. In addition, these minimum tension values are achieved for lower surfactant concentrations. The length of the chain therefore appears to be a factor that can improve the surfactant properties. On the other hand, at equivalent chain length, acidic single-tailed forms provide lower tension values than their double-tailed counterparts. This second chemical characteristic would also provide better surfactant properties. Considering these characterisations of purified compounds, a first trend seems to be defined highlighting the beneficial influence of the single-tailed structure and the length of the alkyl chain. The most interesting compound therefore seems to be the purified compounds **CO_2_HGalC_18_**.

The products studied further were semi-purified mixtures **C_n_PectC_n_** and **CO_2_HPectC_n_**. They consist only of oil-soluble compounds, i.e., degradation compounds, residual fatty alcohol, galacturonic acid-derived surfactants but also derivatives of neutral compounds in the form of alkyl glycosides. The choice to using these mixtures was adopted to limit the presence of insoluble compounds that could disrupt the measurement with the ring. In order to determine the influence of the by-products, for each experiment, we added a curve showing the concentration that was corrected (traced in dotted lines) taking into account only the actual amount of surfactants derived from galacturonic acid present in the mixture. In addition, the experiments were presented according to their chain length, in order to facilitate the understanding of the results.

**Figure 7:**
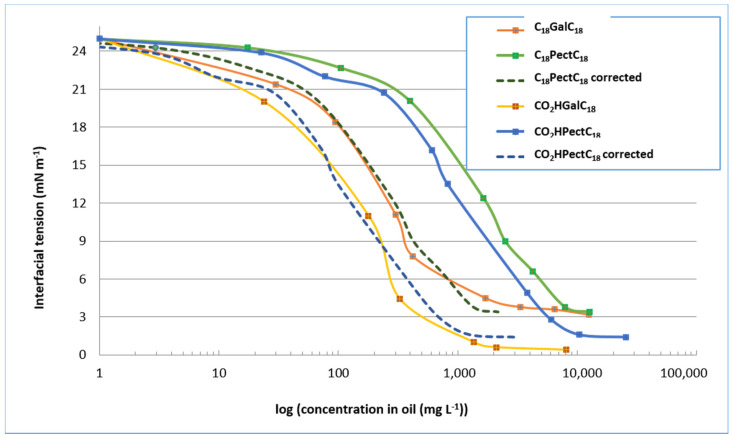
Values of the interfacial tension of surfactant compositions derived from oleic alcohol.

We first examined the surfactant compositions derived from reactions with oleic alcohol. As a reminder, in the case of the semi-purified mixture **C_18_PectC_18_**, the mass proportion of double-tailed compounds **C_18_GalC_18_** is 17%. In the case of the semi-purified mixture **CO_2_HPectC_18_**, the mass proportion of single-tailed compounds **CO_2_HGalC_18_** is 13%. Figure 7 compares the values of the interfacial tensions of purified compounds, products in mixture, but also corrected curves taking into account only the proportion of galacturonic derivatives in these mixtures. If we consider only the interfacial tension curves of the mixed products, we see that the minimum tension values are almost as low as the purified compounds, in the order of 4 mN m^−1^ for the mixture consisting of the double-tailed compound and 2 mN m^−1^ for the mixture consisting of the single-tailed compound. However, these tension values are obtained at different concentrations, which is logically explained by the presence, in semi-purified mixtures, of other compounds (degradation products, fatty alcohol, alkyl glycosides). Indeed, if we consider only the proportion in galacturonic surfactant, we find that these dotted curves are similar to those of the purified compounds. There are rapid decreases in interfacial tensions with, for a concentration of about 1 g L^−1^, values in the order of 3 mN m^−1^ and 6 mN m^−1^ respectively for semi-purified mixtures **CO_2_HPectC_18_** and **C_18_PectC_18_**, identical to purified compounds **C_18_GalC_18_** and **CO_2_HGalC_18_**. This similarity therefore seems to demonstrate that the presence of by-products does little to affect the surfactant properties of compounds derived from oleic alcohol.

**Figure 8:**
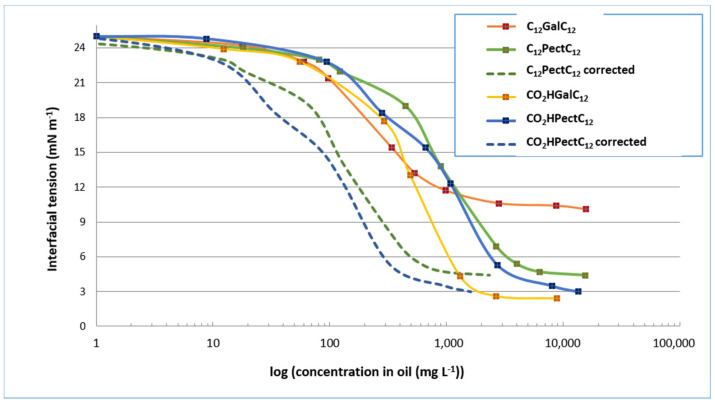
Values of the interfacial tension of surfactant compositions derived from lauric alcohol.

We next explored the surfactant compositions derived from reactions with lauric alcohol. As a reminder, in the case of the semi-purified mixture **C_12_PectC_12_**, the mass proportion in double-tailed compounds **C_12_GalC_12_** is 15%. In the case of the semi-purified mixture **CO_2_HPectC_12_**, the mass proportion of single-tailed compounds **CO_2_HGalC_1_**_2_ is 12%. In comparison with oleic derivatives, the behaviour of compounds derived from lauric alcohol is not as easily comparable. Indeed, for single-tailed structures, the semi-purified compounds **CO_2_HPectC_12_** reduce interfacial tension to values as low as the purified compounds **CO_2_HGalC_12_**, with a minimum value of about 3 mN m^−1^ (Figure 8). On the other hand, the double-tailed forms, in semi-purified mixture **C_12_PectC_12_** provide access to a lower minimum tension than the purified compound, with a value of 5 mN m^−1^ for the mixture compared to 11 mN m^−1^ for the purified compounds **C_12_GalC_12_**. This observation therefore suggests that the presence of the by-products in the mixture may be interpreted as co-surfactants and contributes to the lowering of interfacial tension. In addition, if we consider the corrected curves, we notice, for example, that an interfacial tension of 12 mN m^−1^ is reached for about 150 mg L^−1^ of galacturonic derivative considered in the semi-purified mixtures **C_12_PectC_12_** and **CO_2_HPectC_12_** (dotted curves) versus about 600 mg L^−1^ for purified **CO_2_HGalC_12_**, and 900 mg L^−1^ for purified **C_12_GalC_12_**. The decrease of interfacial tension therefore does not seem to be solely due to the presence of the galacturonic derivative. Although these experiments do not define their role, the by-products have positive influence on the properties at the interfaces and the influence of by-products is different depending on the structure of the surfactant considered. Although the proportions of galacturonic derivatives in mixtures are similar, the presence of by-products does not appear to affect the surfactant properties of oleyl alcohol-derived compounds, while it appears to be beneficial for lauryl alcohol derivatives.

#### 2.3.2. Measurements of Surface Tension

The surface tension measurement requires the compounds to be water soluble, and analyses were therefore carried out on purified surfactants in the form of sodium salts **CO_2_NaGalCn**. These compounds were synthesised quantitatively (see Section Materials and Methods) by neutralising **CO_2_HGalC_12_** and **CO_2_HGalC_18_**, using an aqueous solution of 1N NaOH. The data also include values obtained with anionic surfactants as sodium lauryl ether sulfate (SLES) [35], sodium dodecyl sulfate (SDS) [36] and non-ionic surfactants as alkyl polyglucosides C12 [37], in order to carry out a comparison. Table 4 shows that surface tension—CMC is dependent on the alkyl chain length of galacturonate derivatives. Thus, the shorter the hydrocarbon chain is, the lower the measured surface tension is. In addition, these surface tension values are lower than the reference products. These data also allow us to notice, for the same galacturonate derivatives, that the longer the alkyl chain is, the lower the value of the CMC is. This concentration reaches 3.6 mmol L^−1^ for the oleyl chain compound, a value equivalent to the SDS reference surfactant.

### 2.4. Ecotoxicity Studies

A series of ecotoxicity studies have been performed with the most promising surfactants **CO_2_HGalC_18_**. Experiments to determine the ecotoxicity of products include effects on aquatic organisms, from microalgae to fish. These different pollutant-sensitive organisms serve as controls. Three standardised tests were conducted: an algal growth inhibition test, a microcrustaceous immobilisation test and a lethal toxicity test on a freshwater fish. The microalgae used, called *Pseudokirchneriella subcapitata*, are ubiquitous in the environment and are sensitive to toxic substances. The test is conducted according to the OECD 201 method [38], which corresponds to the percentage of inhibition of the algal growth rate after a 72-h incubation period (CEr50). The microcrustaceans used are Daphnia Magna. They are freshwater crustaceans that are an important nutritional source for many aquatic organisms, their presence in sufficient numbers and in good health helps to maintain a certain balance in their ecosystem. The Daphnia Magna have the distinction of being extremely susceptible to changes, even minor, in the composition of their aquatic environment. The OECD 202 test [39] is based on the determination of the CE50 concentration, which, in 24 h and/or 48 h, immobilises 50% of the microcrustaceans experimented. Finally, the test of freshwater fish, conducted according to the OECD 203 method [40], is to determine the concentration for which the sample has lethal toxicity for 50% of a *Brachydanio rerio* test population after a 96-h exposure period (CL50). *Brachydanio rerio* is one of the model organisms commonly encountered in research laboratories for fish behaviour studies [41]. These tests are based on acute aquatic toxicity tests, i.e., adverse effects on aquatic organisms during short-term exposure. The results observed based on the concentration of the sample to be analysed allow the substances to be categorised into different categories, as shown in Table 5.

Ecotoxicity tests were performed on the purified isomers **CO_2_HGalC_1_**_8_. As shown in Table 6, *n*-oleyl ᴅ-galactosiduronic acids can be considered as non-toxic to the aquatic environment. Indeed, the results on microalgae showed no toxicity at a concentration of 100 mg L^−1^ even by decreasing the population considered. This observation was also valid on microcrustaceans and fish for which no toxicity was visible for a concentration of 100 mg L^−1^. Furthermore, no abnormal behaviour was observed in daphnia and surviving fish at the end of the tests.

### 2.5. Biodegradability Studies

Ready aerobic biodegradability of compounds CO_2_HGalC_18_ was finally studied using the OECD 301 B method [42]. The objective of this test is to determine the release of carbon dioxide by microbial digestion in the aerobic environment of the compound to be analysed. During the test, the compound is placed in a watery medium to which is added a mixed seeding of a plant dealing with urban wastewater. The surfactant studied is the only source of carbon and energy and it is introduced at a theoretical concentration of 10 mg L^−1^ of dissolved organic carbon (COD). The CO_2_ formed during degradation is trapped in external containers. The tests are conducted for 28 days during which the evolution of the biodegradation rate is determined. OECD 301 B method considers a product to be readily biodegradable if the biodegradation rate has reached at least 60% 10 days after the rate has reached 10%. The results revealed a biodegradation rate of 73% after the 10-day interval and 75% after 28 days (Figure S2, Supplementary materials). As the limit of 60% biodegradation was reached, *n*-oleyl ᴅ-galactosiduronic acids CO_2_HGalC_18_ were described as readily biodegradable.

## 3. Discussion

Over the past ten years, intensive research work for the synthesis of non-ionic sugar-based surfactants investigated the direct transformation of natural abundant polysaccharides such as starch, cellulose, hemicellulose or agarose into glycosyl surfactants through the one-pot depolymerisation of these biopolymers into monomers through hydrolysis and/or alcoholysis and their subsequent functionalisation with a lipophilic alkyl chain or through a direct transglycosylation of polysaccharides with fatty alcohols [5,15]. The objective of these approaches was mainly to enhance cost-effectiveness of the process and to provide original surfactant compositions based on hexoses and pentoses. The use of polyuronates as the starting materials for the preparation of both single- and double-tailed nonionic and anionic surfactants is much less common. We have already studied the direct conversion of alginates, i.e., algal polysaccharides exclusively formed of uronic acid units, into uronate or uronamide surfactants [43,44]. To the best of our knowledge, the synthesis of surfactant compositions directly from pectins, has not been described in the literature. One of the major interests for this approach lies in the possibilities to produce both neutral and anionic alkyl galacturonates in addition to alkyl glycosides in a pure form or as mixtures with potential synergistic effects between their constituents.

Our results confirm that pectin is an attractive starting material that can be efficiently depolymerised into monomers despite its complex molecular structure and its high molecular weight. Controlled acid hydrolysis and butanolysis followed by transesterification/transacetalisation processes in fatty alcohols produced major double-tailed galacturonates surfactants and minor alkyl glycosides. An additional saponification step led to the formation of single-tailed alkyl D-galacturonates under sodium salt or acid forms. The obtained results clearly show that the development of one-pot and cascade mode processes contributes to increasing the overall yields as compared to approaches based on successive reactions carried out in a separate mode. Indeed, overall yields for the synthesis of double- or mono-tailed surfactants are twice that of the first stage of the process (hydrolysis/butanolysis of pectin). More surprisingly, the yield relative to the manufacturable process for the production of *n*-oleyl ᴅ-galactosiduronic acids **CO_2_HGalC_18_** was found to be three times higher than that of the synthesis of *n*-butyl (*n*-butyl ᴅ-galactosiduronates) **C_4_GalC_4_**. It could therefore be hypothesised that the one-pot mode and the scale-up maximise the depolymerisation step, by allowing hydrolysis and/or alcoholysis reactions to continue during the transesterification/transglycosylation step.

Using different physico-chemical evaluations and particularly interfacial and surface tension measurements, we have observed that single-tailed structures have better characteristics than their double-tailed counterparts, in terms of surfactant properties. This observation can be justified by the presence of carboxylic acid function at the level of the polar head that brings hydrophilicity to the surfactant structure and thus keeps the hydrophilic–lipophilic balance optimal. In this context, we observe the neat difference in behaviour between the lauryl and oleyl alcohol derivatives. Indeed, the chain length between the two differs for six methylene units. The results obtained confirm that oleyl single or double-tailed compounds decrease the interfacial tension to reach minimum values lower than their equivalents containing lauryl alkyl chains. Regarding the influence of the by-products present in the mixtures, using the interfacial tension measurements, we noticed that these constituents appear to be able to behave as good co-surfactants, or dispersants, and can improve greatly the surfactant properties. However, further tests are needed to determine the role and function of each of the constituents. The presence of co-products in controlled quantities does not appear to affect the surfactant properties of oleyl alcohol-derived compounds, while it has positive effect for lauryl alcohol derivatives. Anyway, the acidic forms are the most interesting, which we interpret in terms of their increased polarity compared to alcohol forms.

To discuss the influence of the structure of the surfactant, we can say that the formation of supramolecular objects in solution, such as micelles, tends to decrease the local entropy of the system. According to the hydrophobic effect, the more lipophilic the molecule, the lower the energy required for micellisation. Thus, the longer the surfactant has a hydrocarbon chain, the more hydrophobic the molecule is likely to be and therefore the lower its CMC, which explains very good surfactant properties. In general, we find that the galacturonate surfactants give similar or better values than standard products used as references. This data can be useful for other areas of application, such as detergency or cosmetology. In addition, unlike most reference compounds, surfactants derived from galacturonic acid have been synthesised from renewable raw materials. In this way, we can expect to have compounds with good eco-compatibility. Indeed, ecotoxicity and biodegradability studies have shown high eco-compatibility of oleyl ᴅ-galactosiduronic acids. In particular, they are not toxic, as evaluated by several standardised tests of ecotoxicity.

## 4. Materials and Methods

### 4.1. Chemistry

Pectins were provided by Cargill (Redon, France) while all other chemicals were commercially available. The commercial oleyl alcohol technical grade contains 85.2% of oleyl alcohol, 8.16% of 1-hexadecanol and 5.96% of 1-octadecanol. All reactions were monitored by thin layer chromatography (Kieselgel 60F_254_ Merck). Compounds were visualised using a H_2_SO_4_ solution (5% H_2_SO_4_ in EtOH and a spatula tip of Orcinol) followed by heating. Geduran 60 (40–63 µm, Merck) was used for column chromatography. NMR spectra were recorded on a Bruker Avance III 400 spectrometer operating at 400.13 MHz for ^1^H, equipped with a BBFO probe with a Z-gradient coil and a GREAT 1/10 gradient unit. The standard temperature was adjusted to 298 K. The *zg30* Bruker pulse program was used for 1D ^1^H NMR, with a TD of 64k, a relaxation delay d1 = 2 s and 8 scans. The spectrum width was set to 18 ppm. Fourier transform of the acquired FID was performed without any apodization in most of the cases. ^13^C NMR spectra were recorded at 100.61 MHz. Several sequences as *jmod*, *dept135* or *zgpg30* were usually used with 64 to 1024 scans depending on the concentration of the sample. TD (total number of data points) was 64k and a relaxation delay of 2 s for a spectral width of 220 ppm was used. Fourier transform was performed after apodization with an exponential function using a LB(line-broadening parameter) of 0.6 Hz. 2D COSY experiments were generally acquired using the *cosygpdqf* pulse program. Matrices consisting of 256–400 (t1) × 2048 (t2) complex data points were recorded; 2 scans were carried out per t1 increment with a 1.5 s recovery delay (d1) and an AQ time of 0.25 s. Processing was performed with a QSINE rsine-squared function in both dimensions (SSB (sine-bell shift) = 0). 2D HSQC (^1^H-^13^C) experiments were acquired using the *hsqcetgpsisp.2* pulse program for high sensitivity with an AQ = 0.25 s, d1= 1.5 s, NS = 2 to 24 depending on the concentration. Generally 256 experiments are acquired (t1). Fourier transform was performed with a QSINE function in both dimensions (SSB: 0). 2D HMBC (^1^H-^13^C) experiments were performed with either the *hmbcplrndqf* or the *impact-hmbc* pulse program. D1 was set to 0.3 s to 1.5 s, NS is dependent on the concentration and the number of series was generally 256 (t1). All the data were processed with a SINE function in both dimensions (SSB = 3). Chemical shifts are mentioned in parts per million (ppm) with tetramethylsilane as an internal standard. Coupling constants were expressed in Hertz (Hz) and the following abbreviations were used to indicate the multiplicity: s (singulet), d (doublet), t (triplet), q (quartet), m (multiplet), dd (doublet of doublets), dt (doublet of triplets) and br (broad signal). Positive or negative ion electrospray (ESI+ and ESI-) mass spectra were acquired on MS/MS TOF and the Shimadzu ensemble LC-MS 2020 mass spectrometers. Biodegradability and ecotoxicity studies were sub-contracted out to Eurofins Company (Maxeville, France) and the conditions used follow the procedures described in references [38,39,40,41,42].

#### 4.1.1. Preparation of *n*-butyl (*n*-butyl-ᴅ-galactoside) uronate **C_4_GalC_4_**

To a suspension of pectins (0.53 g, 1.97 mmol) in water (49 mL, 1000 eq.) was added methanesulfonic acid technical grade 70% wt (0.63 mL, 2.5 eq.). The reaction mixture was heated under reflux at atmospheric pressure for 5 h. *n*-Butanol (25.0 mL, 100 eq.) was then added to the medium and the water was removed by azeotropic distillation using a Dean-Stark apparatus. After water elimination and *n*-butanol recycling, the temperature was cooled to 80 °C and the reaction was allowed to stir for 24 h. The reaction was followed by TLC (CH_2_Cl_2_/MeOH (95/05, *v*/*v*)). The mixture was then neutralised with a 1N NaOH solution and concentrated. The residue was taken up in water and extracted several times by EtOAc. The combined organic layers were dried over MgSO_4_, filtered, concentrated and purified on silica gel (CH_2_Cl_2_, CH_2_Cl_2_/EtOAc (4/1, *v*/*v*), CH_2_Cl_2_/ EtOAc (1/1, *v*/*v*)) to afford **C_4_GalC_4_** (0.46 g, 1.491 mmol, 76%) as a brown oil and butyl 5-(dibutoxymethyl)-2-furoate **5** as a dark brown oil (0.038 g, 0.118 mmol, 6%).

^1^H and ^13^C NMR data of **C_4_GalC_4_** are given in Table S1, Supplementary materials.

Butyl 5-(dibutoxymethyl)-2-furoate, **5:** R_f_ 0.61 (cyclohexane/EtOAc: 90/10, *v*/*v*); ^1^H NMR (400.13 MHz, CDCl_3_): δ 7.11 (d, 1H, *J* = 3.6 Hz, =CH), 6.51 (dd, 1H, *J* = 3.6, 0.8 Hz, =CH), 5.53 (s, 1H, CH(OBu)_2_), 4.29 (t, 2H, *J* = 7.2 Hz, O-CH_2_), 3.60-3.49 (m, 4H, 2 OCH_2_), 1.72 (quin, 2H, *J* = 7.2 Hz, OCH_2_CH_2_), 1.57 (tt, 4H, *J* = 7.2 Hz, 2 OCH_2_CH_2_), 1.44 (tq, 2H, *J* = 7.2 Hz, CH_2_CH_3_), 1.39 (sex, 4H, *J* = 7.2 Hz, 2 CH_2_CH_3_), 0.96 (t, 3H, *J* = 7.2 Hz, CH_3_), 0.91 (t, 6H, *J* = 7.2 Hz, 2 CH_3_); ^13^C NMR (100.61 MHz, CDCl_3_): δ 159.0 (CO_2_CH_2_), 156.3 (=C-CO_2_CH_2_), 144.5 (=C-CH(OBu)_2_), 118.3 (=CH), 110.0 (=CH), 96.4 (CH(OBu)_2_), 66.1 (2 OCH_2_), 64.9 (O-CH_2_), 31.8 (2 OCH_2_CH_2_), 30.9 (OCH_2_CH_2_), 19.5 (2 CH_2_CH_3_), 19.3 (CH_2_CH_3_), 14.0 (2 CH_3_), 13.9 (CH_3_); *m/z* (ESI+) 349.1986 ([M+Na]^+^ requires C_18_H_30_O_5_Na 349.1985).

#### 4.1.2. Preparation of *n*-alkyl (*n*-alkyl-ᴅ-galactoside) uronates **C_n_GalC_n_**

To a suspension of pectins (1 eq.) in water (200 eq.) was added methanesulfonic acid technical grade 70% wt (2.5 eq.). The reaction mixture was heated under reflux at atmospheric pressure for 5 h. *n*-Butanol (100 eq.*)*) was then added to the medium and the water was removed by azeotropic distillation using a Dean-Stark apparatus. After water elimination and *n*-butanol recycling, the temperature was cooled to 80 °C and the reaction was allowed to stir for 24 h. Fatty alcohol (4 eq.) was then added and the reaction was allowed to stir for around 5 h at 70 °C and under reduced pressure (10 mbar); conditions required to remove *n*-butanol. The reaction was followed by TLC (CH_2_Cl_2_/MeOH (95/05, *v*/*v*)). The mixture was then neutralised with a 1N NaOH solution and concentrated. The residue was taken up in water and extracted several times by EtOAc. The combined organic layers were dried over MgSO_4_, filtered, concentrated and purified on silica gel (CH_2_Cl_2_, CH_2_Cl_2_/EtOAc (4/1, *v*/*v*), CH_2_Cl_2_/ EtOAc t (1/1, *v*/*v*)).

*n*-Dodecyl (*n*-dodecyl-ᴅ-galactoside) uronates **C_12_GalC_12._** Hydrolysis of pectins (2.54 g, 9.42 mmol) was achieved in water (47 mL). After reaction with *n*-butanol, 1-dodecanol (11.9 mL) was added and the reaction was allowed to stir for around 5 h at 70 °C and under reduced pressure (10 mbar). After neutralisation with 1N NaOH, the medium was extracted by EtOAc. The combined organic layers were dried over MgSO_4_, filtered and concentrated. The crude was recovered as a mixture (**C_12_PectC_12_**, brown paste, 10.8 g). Purification of the crude mixture on silica gel (CH_2_Cl_2_, CH_2_Cl_2_/ EtOAc (4/1, *v/v*), CH_2_Cl_2_/ EtOAc t (1/1, *v/v*)) afforded a mixture of four isomers (α*f*/βf/β*p*/α*p* = 7/38/9/46, % in mol) **C_12_GalC_12_** (1.65 g, 3.10 mmol, 33% yield, 15% wt) as an ochre powder, degradation products (dark brown liquid, R*f* 0.99, 0.6 g, 5% wt), dodecyl alcohol (slightly yellow paste, 7.98 g, 74% wt) and alkyl glycosides based on hexoses present in pectins (brown oil, R*f* 0.1–0.3, 0.6 g, 6% wt).

^1^H and ^13^C NMR data of **C_12_GalC_12_** are given in Table S1, Supplementary materials.

*n*-Oleyl (*n*-oleyl-ᴅ-galactoside) uronates **C_18_GalC_18._** Hydrolysis of pectins (2.04 g, 7.57 mmol) was achieved in water (38 mL). After reaction with *n*-butanol, oleyl alcohol (13.3 mL) was added and the reaction was allowed to stir for around 5 h at 70 °C and under reduced pressure (10 mbar). After neutralisation with 1N NaOH, the medium was extracted by EtOAc. The combined organic layers were dried over MgSO_4_, filtered and concentrated. The crude was recovered as a mixture (**C_18_PectC_18_**, brown liquid, 12.5 g). Purification of the crude mixture on silica gel (pure CH_2_Cl_2_, CH_2_Cl_2_/EtOAc (4/1, *v*/*v*), CH_2_Cl_2_/EtOAc (1/1, *v*/*v*)) afforded a mixture of four isomers (α*f*/βf/β*p*/α*p* = 10/40/6/44, % in mol) **C_18_GalC_18_** (2.12 g, 3.05 mmol, 40% yield, 17% wt) as a brown oil, degradation products (dark brown liquid, R*f* 0.99, 0.7 g, 6% wt), oleyl alcohol (brown oil, 8.87 g, 71% wt) and alkyl glycosides based on hexoses present in pectins (brown oil, R*f* 0.1–0.3, 0.8 g, 6% wt). An additional flash chromatography on silica gel (CH_2_Cl_2_/ EtOAc/MeOH (20/75/5, *v*/*v*/*v*)) allowed the separation of two oleyl glycosides, oleyl α-ʟ-rhamnopyranoside (**α-ʟ-Rham*p***) and oleyl α-ᴅ-galactopyranoside (**α-ᴅ-Gal*p***).

^1^H and ^13^C NMR data of **C_18_GalC_18_** are given in Table S1, Supplementary materials.

Oleyl α-ʟ-rhamnopyranoside (**α-ʟ-Rham*p***). R_f_ 0.28 (CH_2_Cl_2_/MeOH: 95/5, *v*/*v*); ^1^H NMR (400.13 MHz, CDCl_3_): δ 5.41–5.29 (m, 1H, CH=CH), 4.77 (br, 1H, H-1), 3.92 (br, 1H, H-2), 3.77 (m, 1H, H-3), 3.65 (dt, *J* = 9.5, 6.8 Hz, OCHH), 3.64 (br, 1H, H-5), 3.43 (br, 1H, H-4), 3.42 (dt, *J* = 9.5, 6.8 Hz, OCHH), 2.07-1.94 (m, 4H, CH_2_-CH=CH-CH_2_), 1.65-1.47 (m, 2H, OCH_2_CH_2_),-1.37-1.19 (m, 25H, CH_3_, 11 CH_2_), 0.88 (t, 3H, *J* = 6.7 Hz, CH_3_); ^13^C NMR (100.61 MHz, CDCl_3_): δ 129.9 (2 CH=), 99.5 (C-1), 73.9 (C-4), 71.9 (C-3), 71.2 (C-2), 68.1 (C-5), 67.8 (OCH_2_), 32.7, 31.9, 30.4, 30.3, 30.2, 29.8, 29.6, 29.5, 28.2, 27.2 (14 CH_2_), 14.1 (CH_3_); *m*/*z* (ESI+) 437.20 ([M+Na]^+^ requires C_24_H_46_O_5_Na 437.62).

Oleyl α-ʟ-galactopyranoside (**α-ᴅ-Gal*p***). R_f_ 0.13 (CH_2_Cl_2_/MeOH: 95/5, *v*/*v*); ^1^H NMR (400.13 MHz, CDCl_3_): δ 5.26–5.44 (m, 1H, CH=CH), 4.94 (d, 1H, *J* = 2.4 Hz, H-1), 4.10 (br, 1H, H-4), 3.88–3.85 (m, 4H, H-2, H-3, CH_2_OH), 3.84–3.79 (m, 1H, H-5), 3.70 (dt, 1H, *J* = 9.6, 6.9 Hz, OCHH), 3.45 (dt, 1H, *J* = 9.6, 6.4 Hz, OCHH), 2.08-1.91 (m, 4H, CH_2_-CH=CH-CH_2_), 1.70–1.50 (m, 2H, OCH_2_CH_2_), 1.42–1.16 (m, 22H, 11 CH_2_), 0.88 (t, 3H, *J* = 6.9 Hz, CH_3_); ^13^C NMR (100.61 MHz, CDCl_3_): δ 130.1, 129.8 (2 CH=), 98.6 (C-1), 71.2 (C-5), 70.4 (C-4), 69.7, 69.5 (C-2, C-3), 68.7 (OCH_2_), 62.8 (C-6), 32.1, 32.0, 29.9, 29.8, 29.7, 29.6, 29.5, 29.4, 27.4, 26.3, 22.8 (14 CH_2_), 14.3 (CH_3_); *m*/*z* (ESI+) 453.10 ([M+Na]^+^ requires C_24_H_46_O_6_Na 453.62).

#### 4.1.3. Preparation of *n*-Alkyl-ᴅ-galactosiduronic acids **CO_2_HGalC_n_**

To a suspension of pectins (1 eq.) in water (200 eq.) was added methanesulfonic acid technical grade 70% wt (2.5 eq.). The reaction mixture was heated under reflux at atmospheric pressure for 5 h. *n*-Butanol (100 eq.)) was then added to the medium and the water was removed by azeotropic distillation using a Dean-Stark apparatus. After water elimination and *n*-butanol recycling, the temperature was cooled to 80 °C and the reaction was allowed to stir for 24 h. Fatty alcohol (4 eq.)) was then added and the reaction was allowed to stir for around 5 h at 70 °C and under reduced pressure (10 mbar), conditions required to remove *n*-butanol. The reaction was followed by TLC (CH_2_Cl_2_/MeOH (95/05, *v*/*v*)). A 1N NaOH solution (3.6 eq.) was then added and the reaction mixture was stirred at 30 °C overnight. The mixture was taken up in diethyl ether and washed several times with water. The combined aqueous layers were then acidified with a 4N HCl solution until pH 2, and extracted several times by EtOAc. The combined organic layers were dried over MgSO_4_, and concentrated.

*n*-Dodecyl ᴅ-galactosiduronic acids **CO_2_HGalC_12._** From pectins (2.74 g, 10.2 mmol), 1-dodecanol (12.8 mL), purification by column chromatography on silica gel (CH_2_Cl_2_, CH_2_Cl_2_/AcOEt (4/1, *v*/*v*), CH_2_Cl_2_/AcOEt (1/1, *v*/*v*)) of the diethyl ether layer afforded a mixture of degradation products (dark brown liquid, R*f* 0.99, 0.6 g, 5% wt), dodecyl alcohol (slightly yellow paste, 8.97 g, 77% wt) and alkyl glycosides (brown oil, R*f* 0.1-0.3, 0.6 g, 6% wt). The aqueous layer was acidified with a 4N HCl solution until pH 2, and extracted several times by EtOAc. The combined organic layers were dried over MgSO_4_, filtered and concentrated. This treatment furnished a mixture of isomers (βf/β*p*/α*p* = 13/11/76, % in mol) **CO_2_HGalC_12_** (1.38 g, 3.79 mmol, 37% yield) as an ocher powder. These isomers were added to the by-products in order to form the semi-purified mixture **CO_2_HPectC_12_**, (slightly yellow solid, 11.6 g).

^1^H and ^13^C NMR data of **CO_2_HGalC_18_** are given in Table S2, Supplementary materials.

*n*-Oleyl ᴅ-galactosiduronic acids **CO_2_HGalC_18._** From pectins (2.69 g, 9.98 mmol), oleyl alcohol (21.9 mL), purification by column chromatography on silica gel (CH_2_Cl_2_, CH_2_Cl_2_/AcOEt (4/1, *v*/*v*), CH_2_Cl_2_/AcOEt (1/1, *v*/*v*)) of the diethyl ether layer afforded a mixture of degradation products (dark brown liquid, R*f* 0.99, 0.9 g, 5% wt), oleyl alcohol (brown liquid, 8.97 g, 77% wt) and alkyl glycosides (brown oil, R*f* 0.1-0.3, 0.8 g, 5% wt). The aqueous layer was acidified with a 4N HCl solution until pH 2, and extracted several times by EtOAc. The combined organic layers were dried over MgSO_4_, filtered and concentrated. This treatment furnished a mixture of isomers (βf/β*p*/α*p* = 9/19/72, % in mol) **CO_2_HGalC_18_** (1.85 g, 4.16 mmol, 42% yield) as an ocher powder. These isomers were added to the by-products in order to form the semi-purified mixture **CO_2_HPectC_18_**, (brown liquid, 16.8 g).

^1^H and ^13^C NMR data of **CO_2_HGalC_18_** are given in Table S2, Supplementary materials.

#### 4.1.4. One-Pot Manufacturable Process of Producing **CO_2_HGalC_18_**

In a 5 L reactor equipped with a reflux condenser, pectin (85 g, 0.315 mol) was added to a solution of water (1.6 L.) and methanesulfonic acid technical grade 70% (137.72 g, 1.003 mol, 3.2 eq.). The reaction mixture was heated under reflux for 5 h. *n*-Butanol (2.35 L) was added and the water was removed using a Dean-Stark apparatus. After stirring for 24 h, the heating was stopped and the reaction mixture was transferred into a 20 L round-bottom flask. Oleyl alcohol tech. 80–85% (476 g, 5.6 eq.) was then added and the reaction was allowed to stir in a rotary evaporator (Laborota 20 control) at 70 °C under reduced pressure (16 mbar). After removal of *n*-butanol (4 h), a 1N aq. NaOH solution (1.58 L, 5 eq.) was added to perform the saponification reaction of the ester and the mixture was stirred at 70 °C for 5 h and then at room temperature overnight. *n*-Butanol (2.9 L) was added and water was removed by azeotropic distillation. The resulting mixture was treated with acetone (1 L) at 60 °C and filtered. The acetone addition followed by filtration of the solid was replicated 8–14 times. The solid was subjected to 5N HCl acidification (addition of 0.7 L of water and 172 mL of 5N HCl). Water was eliminated by an azeotropic distillation with methylisobutylketone (4.1 L) that was then recycled. The solid was finally treated with acetone (5 L) and the mixture was filtered using a sintered glass filter of porosity 3. The filtrate was concentrated to provide the isomeric mixture (βf/β*p*/α*p* = 19/13/68, % in mol) **CO_2_HGalC_18_** in a 62% yield.

^1^H and ^13^C NMR data of **CO_2_HGalC_18_** are given in Table S2, Supplementary materials.

#### 4.1.5. Preparation of *n*-Alkyl-ᴅ-galactosiduronic acid sodium salts **CO_2_NaGalC_n_**

*n*-Dodecyl-ᴅ-galactosiduronic acid sodium salts **CO_2_NaGalC_12._** To a suspension of *n*-dodecyl-ᴅ-galactosiduronic acids (0.61 g, 1.683 mmol) in water was added 1N NaOH solution until pH 7. The resulting mixture was freeze-dried to provide **CO_2_NaGalC_12_** (0.65 g, 100% yield) as a white powder. Due to micellisation phenomenon in D_2_O, only the major form of isomers was observed by NMR. ^1^H NMR (400.13 MHz, D_2_O + MeOD(1/1, *v*/*v*) calibrated on MeOD at 3.31 ppm): δ 4.91 (br, 1H, H-1), 4.46 (br, 1H, H-4), 4.17 (br, 1H, H-5), 3.83–3.77 (m, 2H, H-2, H-3), 3.78–3.63 (m, 1H, OCHH), 3.56–3.45 (m, 1H, OCHH), 1.70–1.50 (m, 2H, OCH_2_CH_2_), 1.39–1.18 (m, 18H, 9 CH_2_), 0.90 (t, 3H, *J* = 6.6 Hz, CH_3_); ^13^C NMR (100.61 MHz, D_2_O + MeOD(1/1, *v*/*v*) calibrated on MeOD at 3.31 ppm): δ 175.4 (CO_2_Na), 99.6 (C-1), 71.7 (C-5), 71.6 (C-4), 70.5 (C-3), 69.5 (OCH_2_), 68.8 (C-2), 33.0, 29.9, 29.8, 29.6, 29.5, 29.4, 27.9, 26.5, 22.9 (10 CH_2_), 14.6 (CH_3_). *m*/*z* (ESI-) 361.05 ([M-H]^−^ requires C_24_H_46_O_6_Na 362.16).

*n*-Oleyl-ᴅ-galactosiduronic acid sodium salts **CO_2_NaGalC_18._** To a suspension of *n*-dodecyl-ᴅ-galactosiduronic acids (18.6 g, 41.88 mmol) in water was added 1N NaOH solution until pH 7. The resulting mixture was freeze-dried to provide **CO_2_NaGalC_12_** (19.5 g, 100% yield) as an ochre powder. Due to micellisation phenomenon in D_2_O, only the major form of isomers was observed by NMR. ^1^H NMR (400.13 MHz, D_2_O + MeOD(1/1, *v*/*v*) calibrated on MeOD at 3.31 ppm): δ 5.43–5.27 (m, 1H, CH=CH), 4.91 (br, 1H, H-1), 4.24 (br, 1H, H-4), 4.19 (br, 1H, H-5), 3.87–3.75 (m, 2H, H-2, H-3), 3.74-3.59 (m, 1H, OCHH), 3.56–3.43 (m, 1H, OCHH), 2.11–1.92 (m, 4H, CH_2_-CH=CH-CH_2_), 1.69–1.47 (m, 2H, OCH_2_CH_2_), 1.42–1.14 (m, 22H, 11 CH_2_), 0.87 (t, 3H, *J* = 6.4 Hz, CH_3_); ^13^C NMR (100.61 MHz, D_2_O + MeOD(1/1, *v*/*v*) calibrated on MeOD at 3.31 ppm): δ 175.6 (CO_2_Na), δ 130.7 (2 CH=), 99.3 (C-1), 72.3 (C-5), 71.8 (C-4), 70.7 (C-3), 69.0 (OCH_2_), 68.9 (C-2), 32.2, 32.1, 29.9, 29.8, 29.7, 29.6, 29.5, 28.0, 26.4, 22.9 (14 CH_2_), 14.1 (CH_3_). *m/z* (ESI-) 443.25 ([M-H]^−^ requires C_24_H_46_O_6_Na 444.88).

### 4.2. Physico-Chemistry

Critical micelle concentrations (CMC) and interfacial tensions (IFT) were measured on a force tensiometer Krüss K100. The critical micelle concentrations (CMC) were determined using the *du Noüy* ring method and the *Krüss Laboratory Desktop* software with the *Surfactant Characteristics* program. A water solution of surfactant was prepared around 10 g/L. Only 10 mL of this solution was used for the CMC determination. The deionised water was added thanks to an automatic burette controlled by the software. The concentration and the tension determination were automatically determined by the software. The CMC was measured at 25 °C.

The interfacial tension was determined using the *du Noüy* ring method and the *Krüss Laboratory Desktop* software with the *Surface and Interfacial Tension* program. The method used was the ‘ring tear-off’ mode (e.g., ASTM D971). Instead of detecting the force maximum when the lamella is stretched, the lamella is overstretched until it detaches. The measure is determined at the interface of the two phases. In each case, 35 mL of each phase were previously prepared with the eventual surfactant studied. The general process was performed as follows: first, the oily phase was introduced in the tensiometer. The *du Noüy* ring moved down to the surface and measured the surface tension. Then, the oily phase was replaced by the aqueous phase. The ring moved down to the surface, determined the surface tension and then plunged under the surface. The oily phase was gently added with a plastic pipette above the aqueous phase. The interfacial tension was thus determined automatically by the software at 25 °C.

## 5. Conclusions

This study is the first report describing the cascade mode process for the direct conversion of pectins into *n*-alkyl (*n*-alkyl ᴅ-galactosiduronate) or *n*-alkyl ᴅ-galactosiduronic acid (acid or sodium salt forms) surfactants. Controlled acid hydrolysis and butanolysis reactions efficiently produce *n*-butyl *(n*-butyl ᴅ-galactosiduronate) intermediates, which are subsequently transesterified and transacetalised with fatty alcohols in the same pot and with the same sulfonic acid catalyst. In situ saponification reaction affords the single-tailed *n*-alkyl ᴅ-galactosiduronic acids. This synthesis pathway also leads to the formation of alkyl glycoside by-products that could act as co-surfactants in surface-active compositions. Interestingly, the synthesis of *n*-alkyl ᴅ-galactosiduronic acids has been successfully scaling-up and the reaction/purification conditions have been adapted for potential industrial transposition, even improving the overall yield of the process. Moreover, it is highlighted that these alkyl ᴅ-galactosiduronate molecules in a pure form or as mixtures could exhibit surface activity similar to commercial petro-sourced surfactants. Finally, these surfactants are expected to be eco-friendly molecules as shown with *n*-oleyl ᴅ-galactosiduronic acids, which exhibited both readily biodegradability and absence of aquatic ecotoxicity.

## Figures and Tables

**Table 1:** Reaction conditions and yields (* determined after column chromatography) for the transformation of pectin into *n*-butyl (*n*-butyl ᴅ-galactosiduronates) **C_4_GalC_4._**

Entry	First Step			Second Step	Yields * (%)	
	Hydrolysis	*n* eq. water	*n′* eq. MSA	Reaction Conditions	C_4_GalC_4_	5
**1**	Reflux 5 h	1000	2.5	BuOH 100 eq., reflux 24 h BuOH 100 eq., reflux 48 h BuOH 100 eq., reflux 24 h BuOH 100 eq., 80 °C 24 h BuOH 100 eq., 80 °C 24 h BuOH 50 eq., 80 °C 24 h BuOH 100 eq., 80 °C 24 h	39	27
**2**	Reflux 5 h	1000	2.5		41	36
**3**	Reflux 24 h	1000	2.5		53	24
**4**	Reflux 5 h	1000	2.5		76	6
**5**	Reflux 5 h	200	2.5		21	1
**6**	Reflux 5 h	200	2.5		18	2
**7**	Reflux 5 h	200	1.1		11	3

**Table 2:** Mass composition (* determined by integrating NMR signal for anomeric protons) of the mixtures **C_n_PectC_n_** obtained during the transformation of pectin into *n*-alkyl (*n*-alkyl ᴅ-galactosiduronates) **C_n_GalC_n._**

Compound	Mass Composition (%)				Molar Composition of C_n_GalC_n_ (%) *			
	Apolar Products	Fatty Alcohol	C_n_GalC_n_	Alkyl Glycosides	α*f*	β*f*	α*p*	β*p*
**C_12_PectC_12_**	5	74	15	6	7	38	46	9
**C_18_PectC_18_**	6	71	17	6	10	40	44	6

**Table 3:** Mass composition (* determined by integrating NMR signal for anomeric protons) of the mixtures **CO_2_HPectC_n_** obtained during the transformation of pectin into *n*-alkyl ᴅ-galactosiduronic acids **CO_2_HGalC_n._**

Compound	Mass Composition (%)				Molar Composition of CO_2_HGalC_n_ (%) *			
	Apolar products	Fatty Alcohol	CO_2_HGalC_n_	Alkyl Glycosides	α*f*	β*f*	α*p*	β*p*
**CO_2_HPectC_12_**	5	77	12	6	-	13	76	11
**CO_2_HPectC_18_**	5	77	13	5	-	19	68	13

**Table 4:** CMC and surface tension at the CMC of **CO_2_NaGalCn** and reference surfactants. Standard deviation for the tensiometer is 0.01 N m^−1^.

Compound	𝛄_CMC_ (mN m^−1^)	CMC (mg L^−1^)	CMC (mmol L^−1^)
**Na^+^COO^−^GalC_8_**	23	25.4	77.5
**Na^+^COO^−^GalC_10_**	23	8.0	22.5
**Na^+^COO^−^GalC_12_**	27	7.0	18.2
**Na^+^COO^−^GalC_18_**	32	1.6	3.6
**Sodium Lauryl Ether Sulfate (SLES)**	35	0.3	0.8
**Sodium Dodecyl Sulfate (SDS)**	35	2.4	8.4
**Alkyl Polyglucosides C_12_**	32	-	0.3

**Table 5:** Classification of substances that may have acute toxicity to the aquatic environment.

**Category Acute 1 (very toxic)**	
CL_50_ 96 h (for the fish)	≤1 mg L^−1^ and/or
CE_50_ 48 h (for the shellfish)	≤1 mg L^−1^ and/or
CEr_50_ 72 h (for the seaweed)	≤1 mg L^−1^
**Category Acute 2 (toxic)**	
CL_50_ 96 h (for the fish)	>1 but ≤10 mg L^−1^ and/or
CE_50_ 48 h (for the shellfish)	>1 but ≤10 mg L^−1^ and/or
CEr_50_ 72 h (for the seaweed)	>1 but ≤10 mg L^−1^
**Category Acute 3 (poorly toxic)**	
CL_50_ 96 h (for the fish)	>10 but ≤100 mg L^−1^ and/or
CE_50_ 48 h (for the shellfish)	>10 but ≤100 mg L^−1^ and/or
CEr_50_ 72 h (for the seaweed)	>10 but ≤100 mg L^−1^
**Above 100 mg L^−1^, the substance is considered as non-toxic**	

**Table 6:** Results of ecotoxicity tests on **CO_2_HGalC_1_**_8._

Test	Effect	Toxicological Descriptor *	CO_2_HGalC_18_
**Microalgae**	Rate of growth	CEr_50_-72 h	Non-toxic at 100 mg L^−1^
		CEr_20_-72 h	Non-toxic at 100 mg L^−1^
		CEr_10_-72 h	Non-toxic at 100 mg L^−1^
		NOEC-72 h	≥100 mg L^−1^
**Daphnia**	Immobilisation	CE_50_-24 h	Non-toxic at 100 mg L^−1^
		CE_50_-48 h	Non-toxic at 100 mg L^−1^
**Fish**	Mortality	CL_50_-96 h	Non-toxic at 100 mg L^−1^

* The toxicological descriptors C X-T correspond to the concentrations causing an effect on X % of the population after a time T. NOEC (No observed effect concentration) is the highest concentration in the range of tests performed that do not cause significant effects on organisms.

## Data Availability

All data are contained within the article and the Supplementary Materials.

## Supplementary Materials

The following are available online. Figure S1: HPSEC analysis of pectin. Figure S2: Biodegradability results (**CO_2_HGalC_188_**) according to the OCDE 301 B method. Table S1: ^1^H and ^13^C NMR data (CDCl_3_, 400.13, 100.61 MHz) for isomers **C_n_GalC_n_**. Table S2: ^1^H and ^13^C NMR data (CDCl_3_, 400.13, 100.61 MHz) for isomers **CO_2_HGalC_n_**.

## Sample Availability

Samples of the compounds are not available from the authors.
