# Supplementary material for: Transformation of Pectins into Non-Ionic or Anionic Surfactants Using a One-Pot and Cascade Mode Process
Source: Molecules. 2021 Mar 31;26(7):1956. doi: 10.3390/molecules26071956 (PMC8036317; doi:10.3390/molecules26071956)
Supplement: Supplementary file 1 [file molecules-26-01956-s001.pdf]

Supplementary Materials :

Transformation of Pectins into Nonionic or Anionic Surfactants Using a One-Pot and Cascade Mode Process

Damien Milliasseau, Jelena Jeftić, Freddy Pessel, Daniel Plusquellec and Thierry Benvegnu

Contents:

**Figure S1**	HPSEC analysis of pectin.	2
**Figure S2**	Biodegradability results (**CO_2HGalC_{18}**) according to the OCDE 301 B method	3
**Table S1**	^{1}H and ^{13}C NMR data ($CDCl_3$, 400.13, 100.61 MHz) for isomers **C_nGalC_n**.	4-5
**Table S2**	^{1}H and ^{13}C NMR data ($CDCl_3$, 400.13, 100.61 MHz) for isomers **CO_2HGalC_n**.	6

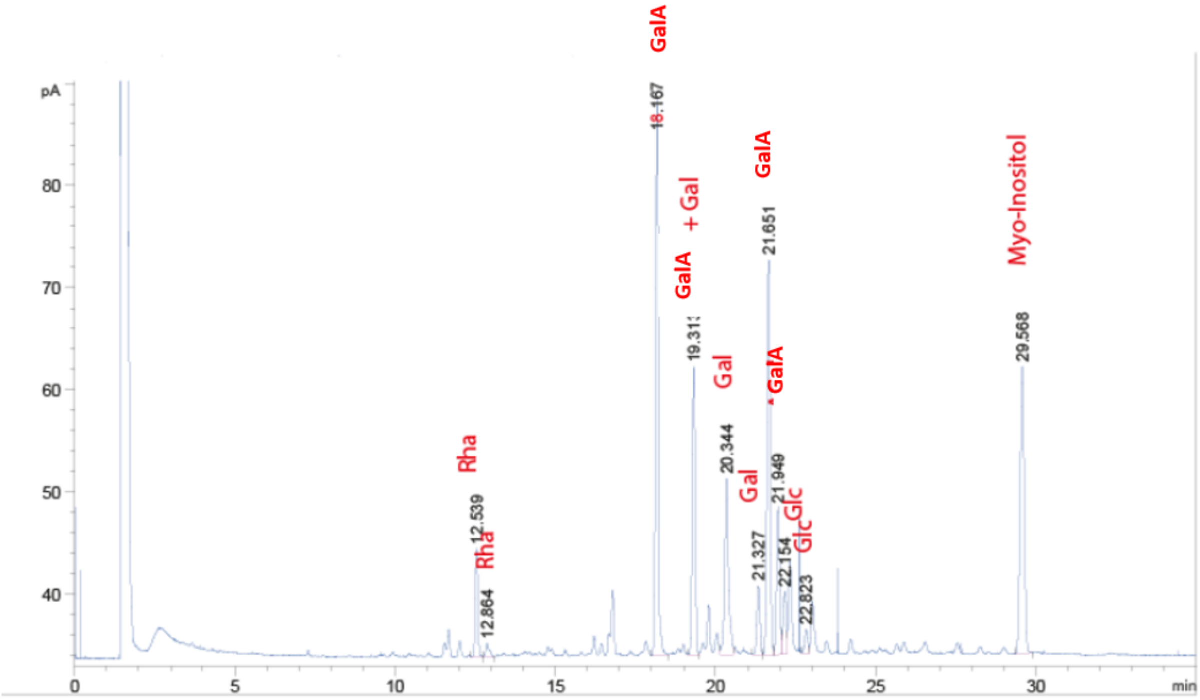

GalA: D-Galacturonic acid; **Rha**: L-Rhamnose; **Gal**: D-Galactose; **Glc**: D-Glucose

Figure S1: HPSEC analysis of pectin.

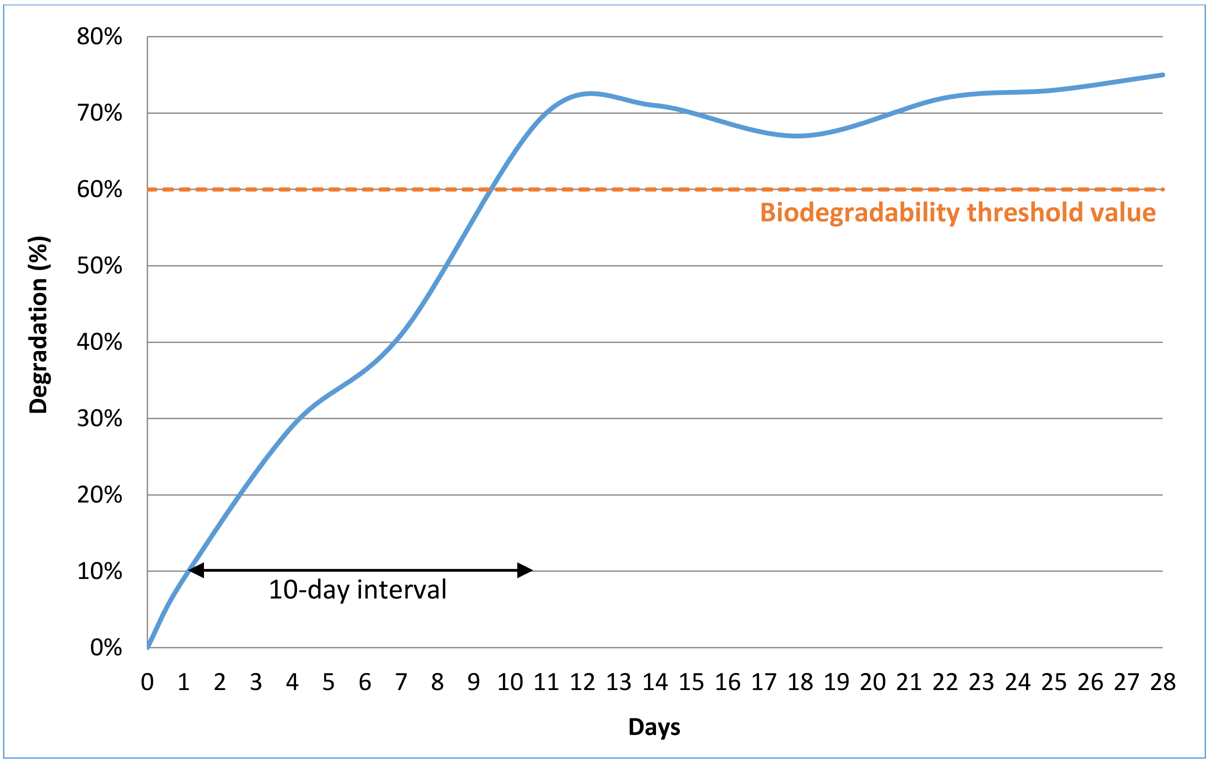

Figure S2: Biodegradability results (**CO_2HGalC_{18}**) according to the OCDE 301 B method.

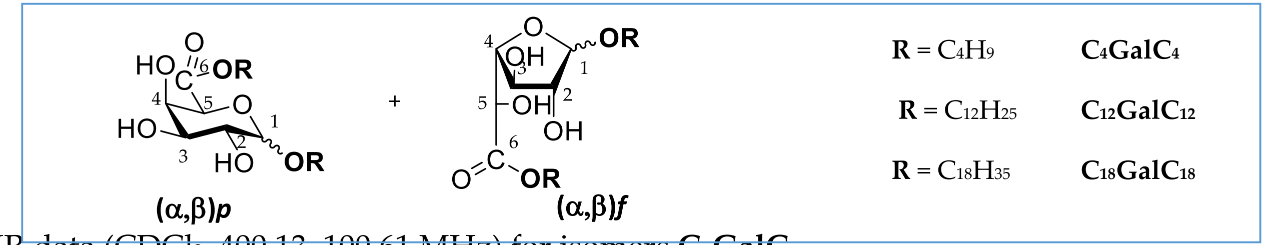

Table S1: ^{1}H and ^{13}C NMR data ($CDCl_3$, 400.13, 100.61 MHz) for isomers **C_nGalC_n**.

δ (ppm)		1	2	3	4	5	6	OCH₂	CO₂CH₂	(CH₂)ₙCH₃	CH=CH
**C_4GalC_4 α*f***	**[1]H**	4.90 d, *J* = 5.0 Hz	4.08	4.35	4.17	4.24	-	3.47 / 3.81	4.22 / 4.37	1.62-0.84	-
	**[13]C**	100.96	78.37	75.88	82.90	70.15	172.34	69.16	65.84	31.59-13.64	-
**C_4GalC_4 β*f***	**[1]H**	5.00 s	3.96 d, *J* = 11.3 Hz	4.05 d, *J* =11.4 Hz	4.45 d, *J* = 1.8 Hz	4.40	-	3.34 / 3.57	4.20 / 4.36	1.65-0.84	-
	**[13]C**	108.73	78.52	78.34	87.62	70.35	172.15	67.58	66.66	31.48-13.56	-
**C_4GalC_4 β*p***	**[1]H**	4.23 d, *J* = 7.4 Hz	4.37 d, *J* = 1.5 Hz	3.66	4.20	4.08	-	3.51 / 3.93	4.17	1.64-0.84	-
	**[13]C**	103.02	70.43	73.20	70.11	74.10	168.34	70.28	65.49	31.49-13.61	-
**C_4GalC_4 α*p***	**[1]H**	4.94 d, *J* = 3.1 Hz	3.83	4.23	4.33 d, *J* = 1.5 Hz	3.93	-	3.43 / 3.66	4.14	1.64-0.86	-
	**[13]C**	98.87	68.45	70.51	70.34	70.03	169.24	68.64	65.37	31.42-13.61	-
**C_{12}GalC_{12} α*f***	**[1]H**	4.87 d, *J* = 4.9 Hz	4.06 dd, *J* = 7.6, 4.9 Hz	4.31	4.15	4.29	-	3.46 / 3.74	4.16 / 4.25	1.68-0.87	-
	**[13]C**	100.95	78.02	74.85	82.92	69.92	172.23	69.25	65.70	31.90-13.99	-
**C_{12}GalC_{12} β*f***	**[1]H**	4.97 s	3.96	4.09 d, *J* =2.0 Hz	4.39 d, *J* =1.7 Hz	4.41	-	3.39 / 3.63	4.17 / 4.32	1.72-0.86	-
	**[13]C**	108.51	78.82	78.11	87.23	70.24	172.12	67.90	66.69	32.01-14.20	-
**C_{12}GalC_{12} β*p***	**[1]H**	4.23 d, *J* = 7.4 Hz	4.39	3.68	4.27	4.13 d, *J* = 1.4 Hz	-	3.51 / 3.98	4.16	1.70-0.87	-
	**[13]C**	103.02	71.06	73.25	70.63	74.12	168.22	70.00	65.89	31.02-14.18	-
**C_{12}GalC_{12} α*p***	**[1]H**	5.04 br	3.84	4.34	4.40 d, *J* =1.5 Hz	3.84	-	3.51 / 3.75	4.22	1.72-0.88	-
	**[13]C**	98.64	69.37	70.17	70.22	70.93	168.84	69.26	66.01	32.07-14.27	-
**C_{18}GalC_{18}**	**[1]H**	4.89	4.06	4.37	4.18	4.24	-	3.46 / 3.79	4.18 / 4.32	2.05-0.87	5.39-5.32

α*f*		d, *J* = 5.0 Hz	dd, *J* = 7.4, 5.0 Hz			d, *J* = 2.1 Hz					
	^{13}C	100.94	78.35	75.73	82.90	70.11	172.34	69.45	66.09	32.03-14.18	130.17-129.80
**C$_{18}$GalC$_{18}$ β*f***	^{1}H	4.99 s	3.96 s	4.09	4.44 d, *J* =1.6 Hz	4.40 d, *J* =1.6 Hz	-	3.40 / 3.66	4.20 / 4.35	2.06-0.87	5.38-5.31
	^{13}C	108.71	78.55	78.33	87.59	70.36	172.12	67.92	66.90	32.75-14.25	130.17-129.80
**C$_{18}$GalC$_{18}$ β*p***	^{1}H	4.27 d, *J* =7.3 Hz	4.39	3.70	4.26	4.13 d, *J* =1.6 Hz	-	3.55 / 3.99	4.21	2.08-0.87	5.36-5.31
	^{13}C	102.99	71.36	73.25	70.63	74.12	168.17	69.92	65.97	32.05-14.20	130.03-129.79
**C$_{18}$GalC$_{18}$ α*p***	^{1}H	4.99 d, *J* = 2.9 Hz	3.86	4.28	4.36	3.86	-	3.47 / 3.68	4.18	2.06-0.86	5.34-5.30
	^{13}C	98.90	68.79	70.49	70.40	70.49	169.14	69.13	65.82	32.70-14.19	130.03-129.79

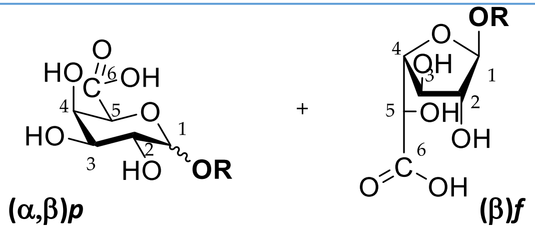

R = $C_{12}H_{25}$ **CO_2HGalC_{12}**

R = $C_{18}H_{35}$ **CO_2HGalC_{18}**

δ (ppm)		1	2	3	4	5	6	OCH_2
**CO_2HGalC_{12}** **β*f***	**^{1}H**	4.87	3.96	4.12	4.21	4.25	-	3.40 / 3.65
	**^{13}C**	109.36	83.32	77.90	84.77	70.24	175.48	69.04
**CO_2HGalC_{12}** **β*p***	**^{1}H**	-	-	-	-	-	-	-
	**^{13}C**	105.38	72.74	75.32	72.17	76.05	172.78	71.88
**CO_2HGalC_{12}** **α*p***	**^{1}H**	4.89	3.79	3.80	4.23	4.39	-	3.52 / 3.69
	**^{13}C**	100.63	69.62	70.98	71.85	71.76	172.59	69.86
**CO_2HGalC_{18}** **β*f***	**^{1}H**	4.85	3.94	4.13	4.24	4.26	-	3.40 / 3.66
	**^{13}C**	109.40	83.32	77.96	84.84	70.30	175.52	69.06
**CO_2HGalC_{18}** **β*p***	**^{1}H**	4.28	3.54	3.54	4.17	4.20	-	3.55 / 3.96
	**^{13}C**	104.45	72.03	74.51	71.46	75.27	172.08	71.17
**CO_2HGalC_{18}** **α*p***	**^{1}H**	4.90	3.81	3.81	4.25	4.39	-	3.50 / 3.69
	**^{13}C**	100.63	69.64	71.01	71.87	71.80	172.64	69.89

Table S2: ^{1}H and ^{13}C NMR data ($CDCl_3$, 400.13, 100.61 MHz) for isomers **CO_2HGalC_n**.
